# Perceptions of physiotherapy clinical educators’ dual roles as mentors and assessors: Influence on teaching–learning relationships

**DOI:** 10.4102/sajp.v75i1.468

**Published:** 2019-03-28

**Authors:** Ilse S. Meyer, Alwyn Louw, Dawn Ernstzen

**Affiliations:** 1 Centre for Health Professions Education, Faculty of Medicine and Health Sciences, Stellenbosch University, Cape town, South Africa; 2 Division of Physiotherapy, Department of Health and Rehabilitation Sciences, Faculty of Medicine and Health Sciences, Stellenbosch University, Cape town, South Africa

## Abstract

**Background:**

Central to clinical education is the teaching–learning (T-L) relationship that evolves between the clinical educator (CE) and the student. Within this T-L relationship, CEs may be expected to fulfil dual roles as mentors and assessors of students. Challenges for both parties may arise when CEs take on these different roles.

**Objectives:**

The goal of this study was to ascertain how CEs perceived the influence of their dual roles as mentors and assessors on their T-L relationships with physiotherapy students.

**Method:**

Individual interviews were semi-structured with nine CEs during this qualitative descriptive study at the Division of Physiotherapy, Faculty of Medicine and Health Sciences, Stellenbosch University. A content analysis followed to analyse the data obtained. An iterative process, aimed to understand the phenomena under study, was conducted via an interpretive approach in context. This revealed main themes that were identified and refined.

**Results:**

Clinical educators experienced challenges when their role changed from being a mentor to that of assessor. These challenges affected the learning of students, as they influenced the T-L relationship. Clinical educators experienced ambiguities regarding their dual roles and, as a result, their expectations were often not fulfilled.

**Conclusion:**

Students’ learning processes were negatively affected by the changing roles of CEs, who acted as mentors and later as assessors of clinical competence during the students’ clinical rotations. The positioning theory was offered as a framework to resolve the challenges created by the dual roles and to manage expectations between CEs and students. The T-L encounters could be enhanced if students and CEs aligned themselves to a learning-centred paradigm where the focus was on learning and where the needs of the diverse students and the expectations of CEs were balanced. Further research should explore how a workshop with role play, demonstrating to CEs in practice how to reposition themselves, would impact the relationships between both parties.

**Clinical implications:**

It is essential to ensure a positive T-L relationship between a CE and a student as this will improve the quality of learning in the clinical environment and, therefore, directly influence student’s patient management. Implementing faculty development programmes to address this, should be further explored.

**Keywords:**

physiotherapy; clinical education; teaching–learning relationship; learning-centred; dual roles.

## Introduction

The clinical environment is a robust context for clinical educators (CEs) to support students’ clinical education (Kilminster et al. 2007). The clinical environment provides authentic experiences for students to be practically involved in the process of learning (Ker, Cantillon & Ambrose 2008).The 4-year Bachelor of Science in Physiotherapy (BSc Physio) undergraduate degree programme at the Division of Physiotherapy, Faculty of Medicine and Health Sciences (FMHS), Stellenbosch University (SU), contains a prominent clinical module, namely Clinical Physiotherapy. This module is regarded as an essential constituent of the degree programme.

During engagement with patients, students not only receive direct supervision from various CEs but are also facilitated by them as they fulfil the many roles of assessor, mentor, role model, advisor, counsellor, teacher and manager, as listed by Harden and Crosby (2000). During engagement with patients, students are guided by a variety of CEs, who act as role models (Cruess, Cruess & Steinert 2008) while providing direct supervision to students. It stands to reason that being a role model necessitates a positive teaching–learning (T-L) relationship for learning to be successful in the clinical environment, as confirmed by Laitinen-Väänänen (2008) and highlighted by Vygotsky and Cole’s (1978) principles of higher psychological processes.

A distinguishing characteristic of Vygotsky’s theories of learning is known as the zone of proximal development (ZPD) (Vygotsky & Cole 1978). This refers to the distance between a student’s ability to perform a task under the guidance of a CE and the student’s ability to solve the problem independently. According to Vygotsky, this zone is where actual learning occurs. To facilitate learning, CEs can, therefore, adjust their teaching strategies through social interactions with the students, according to the students’ progress, that is, ‘scaffolding’ (Tilley et al. 2007). For scaffolding to be used successfully as a learning tool, participants need to share experiences while communicating with one another. This interaction is defined by Vygotsky and Cole (1978) as collaborative dialogue, as students actively participate in communities of practice (Lave & Wenger 1991). In 1999, Wenger confirmed that communities of practice are ‘groups of people who share a concern or a passion for something they do and learn how to do it better, as they interact regularly’ (Wenger 1999). Therefore, communities of practice are present in physiotherapy clinical settings where students and CEs explore what students have already learned and what they still need to learn in a clinical environment.

Furthermore, Vygotsky and Cole (1978) advocate that a ‘psychological symbiosis’ should develop between a CE and a student in order to ensure a positive relationship that could assist students to practise more affectively in a clinical setting. A negative relationship will inhibit students because they will then only perform routine tasks (Newton, Billett & Ockerby 2009). If conflicts are present, the psychological symbiosis will be unsuccessful and the T-L relationship could be negatively influenced (Power & Bogo 2003).

It would be possible for CEs to perform the roles of mentors if they were experiencing symbiotic relationships with their students. Their relationships would be relaxed and friendly. However, it is unclear how the T-L relationship and the learning process are influenced when the mentor’s role changes to that of an assessor. Within the context of the current study, the CE acts as a mentor and facilitator of learning during the first five weeks of the clinical rotation. During the sixth and final week of the orientation, the role of the CE changes, and the same CE has to perform a summative assessment of clinical competence for the student. Ezzat and Maly (2012) maintain that mentorship is a potent means to improve clinical skills.

Summative assessment involves direct observation by CEs during a variety of clinical settings with various patients. Students are expected to demonstrate what they know and have learned, their skills as well as their attitudes, while treating real-life patients. The assessor will determine whether they have met all the required standards and outcomes (Van der Vleuten & Schuwirth 2005). Summative block assessments take place mid-year and two exit clinical examinations at the end of their final year. The latter involve two assessors, namely the CE and an external assessor. Required standards need to be attained according to those determined by the regulatory bodies and the curriculum. The pass or fail status of borderline students is clearly explained to the students according to the set criteria and weighting of the assessments used.

Clinical educators, as assessors, are responsible for ensuring that only competent practitioners enter the physiotherapy profession and are, therefore, accountable to society, professional bodies and to patients, acting as gatekeepers, they declare by their judgements that students are ‘fit for purpose’ (Wass et al. 2001). Neary (2000) states that it is important for assessors to be skilful, trained, fair, and knowledgeable and well prepared. Observation of students’ practices over a wide spectrum of types and needs includes the history-taking and physical examination of new patients or the re-evaluation and treatment suitable for known patients.

The changing of roles may be demonstrably challenging for both CEs and students. Davies and Harré (1990) declare that their positioning theory, which is relevant in the T-L relationship, could be practised to facilitate learning. It could be an extremely dynamic situation if a person’s position or any change in one’s position during communication with others in communities of practice, is clearly understood.

It was important to ascertain how the dual roles of CEs at the Physiotherapy Division, SU, affect the T-L relationships, and from CEs’ viewpoint, as opposed to the views of the students (Meyer, Louw & Erntzen 2017).

The aims of this study were to establish the following:

- how CEs’ dual roles affected the T-L relationships
- to identify possible problem areas in order to offer recommendations to enhance the students’ quality of learning.

The objectives were as follows:

- to ascertain how the CEs interpreted the dual roles they performed, and
- to explain these experiences, giving suggestions on how to optimise the T-L relationship.

## Method

The study engaged in a phenomenological inquiry (Maree 2007), which followed a qualitative research design, with an interpretivist approach. Information was collected from the participants by means of interviews, reviewed and analysed in context. The CEs’ input formed the basis of the investigation.

The population included CEs involved in clinical education for third and fourth years at the time of the study. The sampling for the purpose of individual interviews consisted of nine CEs, two of whom were responsible for the clinical education of third years, six for fourth years and one for both third- and fourth-year students. Clinical educators were selected on the basis of those with experience, namely number of years of supervision, expertise in specific fields, knowledge of adult educational principles, supervision of students during clinical education, as well as some with less experience – all of whom were selected from the division’s database. Their experiences ranged from newly appointed to 20 years of supervision. This ensured that the sampling framework could generate a wider spectrum of information, together with a variety of CEs’ perceptions, knowledge, expertise and experience.

The participants were all female, and the interviews occurred over a period of 2 months. The participants were interviewed individually in the language they preferred, either English or Afrikaans. Written informed consent was received from all the participants, and participation was voluntary. They were informed of the confidential handling of data.

The researcher developed an interview discussion schedule that provided strategies to define the line of enquiry. Questions were discussed by the researcher and interviewer to ensure their efficiency in order to stay focused and cover all important points. The questions were open-ended based on the aims as well as on aspects from the literature that appeared to have had an impact on the T-L relationship. These questions covered the CEs’ general experiences while supervising students, their specific responsibilities and challenges regarding both their mentor and assessor roles.

## Data management and analysis

A digital voice recorder was utilised during the interviews. The recorded interviews were imported onto a computer. They were protected by a password. Each recording was provided with a special serial number and copied onto a flash disk. These recorded interviews were transcribed by an independent assistant, who downloaded them on a secured computer. Member checking of the transcriptions followed.

The data were analysed and interpreted according to the context as described by Miles and Huberman (1994). The first author studied the verbatim transcripts to become acquainted with the contents. The contents were coded according to Microsoft Office OneNote 2007. Patterns that emerged were identified and arranged into themes. These were entered into a codebook. Categories that developed were reviewed and refined. According to Kelly (2009), an iterative process aimed to understand the phenomena of this study was conducted via an interpretive approach in context. Trustworthiness of the findings (Lincoln & Guba 1985) was ensured by checking themes and categories against the transcriptions of the interviews. The results are set out in the following section.

### Ethical considerations

Ethical approval for the study was obtained from the Human Research and Ethical Committee (HREC), Faculty of Medicine and Health Sciences, Stellenbosch University (protocol number: S12/11/289) and from the Institutional Research and Planning Division (IRP) at SU.

## Results

Three main themes were identified, namely:

- *challenges* experienced by CEs pertaining to their dual roles in the T-L relationship
- *expectations* CEs had of the T-L relationship, and
- clinical educators’ *preferences* regarding the dual roles.

### Theme 1: Challenges

Overall the CEs perceived the dual roles as challenging, as they found it demanding to alternate between the roles. The following identified categories will explain the findings in this theme:

*Inconsistencies*: Inconsistencies were experienced by CEs regarding their behaviour and attitudes among themselves when alternating between the dual roles. Inconsistencies were reported to cause confusion among students, which resulted as a negative effect on students’ learning experiences:

‘Not all CEs act the same during assessments. Some ask more questions as the assessment continues, while others intervene more during the process. I am guilty of this … you chip in too quickly instead of distancing yourself while you observe the performance.’ (Participant 8, female, 15 years’ experience)

Some CEs identified inconsistencies as students showed signs of being anxious when they were assessed:

‘During clinical assessments, which students often find stressful, they will do the most peculiar things that they would never do during the clinical block.’ (Participant 4, female, 15 years’ experience)

‘I think you can never get away from the fact that students know you’re going to mark them. There’s always that undercurrent. I’m not sure it facilitates learning well. I think it facilitates them doing what they think you want them to do.’ (Participant 3, female, 20 years’ experience)

*Bias*: The participants reported being extremely aware of the potential for bias that was created by their dual roles. The contrast between the students’ performances during the clinical rotation versus during the assessment was emphasised as a stressor of disparity:

‘It is difficult when you supported the students for weeks and you know their potential ability. It is difficult to then be objective and observe and forget about what happened the past few weeks, especially if the student didn’t perform well.’ (Participant 7, female, 2 years’ experience)

‘I think every assessment is subjective. I think the possibility of subjectivity is there if a student is very good or perceived to be good on several occasions. It could influence the mark, especially for good students that the halo effect could go through.’ (Participant 3, female, 20 years’ experience)

‘If you expect a student, who performed well throughout the clinical block, to do well and he doesn’t, you are inclined to give him credit. And similarly, if a student performs badly throughout the block, and he performs very well during the block assessment, you will tend to be more critical towards him. I, myself, am guilty of that.’ (Participant 8, female, 15 years’ experience)

*Conflict*: Conflict in this context reflected disagreement between the students and CEs. Conflict arose from different situations, such as failing a student, possible perception of intimidation of students, the challenges of proper feedback and diversity challenges.

*Failing*: When CEs had to fail students during clinical performance assessments, potential conflict situations developed, which caused uncertainty within the CE herself, as well as between the CE and the student:

‘This is always very, very difficult. Firstly, we know what the consequences will be when students fail, and that can result in a huge amount of stress. It is usually more difficult to decide which way to go when a student ends up with borderline results… and you think that you should actually give her credit and the benefit of the doubt.’ (Participant 8, female, 15 years’ experience)

‘The first time I failed a student, it had a big impact on our T-L relationship. She was very angry and I was so upset, because I thought it was my fault. It was a very traumatic experience. It did affect our relationship badly, which upset me a lot.’ (Participant 3, female, 20 years’ experience)

*Intimidation*: Clinical educators confirmed that students could feel overwhelmed by the presence of the CE during a patient encounter and during assessment. This feeling of fear was stated as a barrier for students to disclose their learning needs and the issues with which they struggled:

‘Some students can feel intimidated by you. Some students will withdraw.’ (Participant 6, female, 4 years’ experience)

‘I think the students sometimes do not want to reveal their limitations. They tend to distance themselves. I think they don’t want you to observe their shortcomings as they know you will be assessing them at the end of the block.’ (Participant 7, female, 2 years’ experience)

*Feedback*: Clinical educators found giving feedback difficult and challenging, especially when students reacted negatively to the feedback received:

‘The marking is not as difficult as giving feedback. To give face-to-face verbal feedback, that to me is the difficult part.’ (Participant 8, female, 15 years’ experience)

‘It was very difficult to provide them with the feedback of them failing, as it was emotional for both of us. That was challenging to handle.’ (Participant 6, female, 4 years’ experience)

‘The most challenging is when students show aggression and do not understand your comments.’ (Participant 5, female, 16 years’ experience)

*Diversity*: Clinical educators found dimensions of diversity among both parties challenging. This resulted in conflict and influenced the relationships:

‘Sometimes it’s not easy, because of the personality differences of the students.’ (Participant 3, female, 20 years’ experience)

‘It is amazing how different they can react to feedback. It is challenging to work with so many different students and to treat them accordingly.’ (Participant 1, female, 1 year’s experience)

### Theme 2: Expectations

Participants mentioned the lack of effective communication, reciprocal regard for one another and trust in the T-L relationship. It was highlighted by CEs as critical to reach the learning outcomes during clinical rotations. Clinical educators clearly stated their expectations of the T-L relationship as described in the following:

‘I think it is necessary to clear expectations right from the start, what you as a clinical educator can expect from the student and what they can expect from you. There should be a contract in place between you.’ (Participant 2, female, 8 years’ experience)

‘A certain level of trust is needed to show them the right direction.’ (Participant 3, female, 20 years’ experience)

‘The clinical environment influences the learning of students; therefore the environment should be positive, supportive, with positive role models. If students are anxious and intimidated, or not on standard, then they suffer from this emotional roller coaster effect that can affect their learning experiences.’ (Participant 6, female, 4 years’ experience)

### Theme 3: Preferences

The CEs reported that they had specific preferences with regard to the dual roles for which they were responsible.

*Roles*: Some CEs preferred the dual role, even though demanding challenges became evident. Others disagreed:

‘I do feel comfortable in both roles after sixteen years’ experience; however it is not always easy. Sometimes it can be a bit personal. I enjoy the mentor’s role more. You should be able to distance yourself from the mentor role when you assess the student. I think it is difficult to change from mentor to assessor without experience.’ (Participant 5, female, 16 years’ experience)

‘I find it quite difficult and I try to separate the two, although the system as it is at the moment doesn’t allow for that. I can do both roles, but I find it’s easier to do one. Either be the marker, or the mentor, then they are separate issues. It’s a case of putting on a different hat. Both can mediate each other and be balanced. I’d rather be in a mentorship role, but it doesn’t mean that I don’t understand the importance of the professional exit exam …much better and nicer role to be a mentor, but maybe that’s my personality.’ (Participant 3, female, 20 years’ experience)

‘I think it is necessary to be able to function in both roles, as by facilitating students, you will be able to determine the level of the students’ ability and in what area they still need more support.’ (Participant 2, female, 8 years’ experience)

‘I do think the person that’s the mentor should not be the assessor as well. There can be two persons assessing, to allow a more objective view. I think it is impossible to fulfil both roles and should involve two people.’ (Participant 8, female, 15 years’ experience)

*Relationship*: Some CEs preferred to address students in groups, rather than individually. They reported that a one-to-one situation tended to be more personal and difficult:

‘I always try to have them in groups of two or more, so that it is never personal.’ (Participant 3, female, 20 years’ experience)

‘The clinical educator should, however, not become too familiar with the students.’ (Participant 8, female, 15 years’ experience)

One CE mentioned the need for having a good relationship with students to ensure that students felt comfortable to disclose their lack of knowledge:

‘I have a comfortable T-L relationship with students. They need to feel comfortable to approach me to ask questions.’ (Participant 5, female, 16 years’ experience)

## Discussion

This study, the first of its kind in this context, confirmed that the dual roles of the CE strongly affected the T-L relationship and the learning and assessment processes in clinical education in positive and negative ways. It was essential to discuss and address the challenges that the CEs faced in order to minimise the negative effects they could have on the relationships and students’ learning. Inconsistencies, also referred to as *disparities* when CEs were inconsistent in what was taught to students and what was expected from students during the assessment process, were identified. These inconsistencies influenced the validity and reliability of assessment procedures, as Gravett and Geyser (2004) confirmed. The inconsistencies occurred when CEs changed roles and were the dominant factors that influenced the relationship negatively. The dual roles of the CEs were perceived to impede the objectivity of some CEs during assessment. Although some differences of opinion would always arise among CEs, as Dalton, Davidson and Keating (2012) acknowledged, the challenge is to evaluate what level of disagreement should be accepted. Inconsistencies became apparent when the expectations of students and those of CEs differed. There was clearly a lack of what was expected from both roles as mentors and assessors, respectively.

Furthermore, CEs were aware of bias being a major challenge. Biases of assessors could be problematic, especially where observation as an assessment strategy is used. Epstein et al. (2004) consider summative assessments of clinical performances to be an opportunity to demonstrate adequate reliability and validity for its purpose. Biases were also present in some CEs’ judgements. The accuracy of the assessment procedure could therefore be influenced negatively (Borrell-Carrió & Epstein 2004). A negative perception was called the ‘devil effect’, whereas a positive one was perceived as a ‘halo effect’ (Participant 3, female, 20 years’ experience), also referred to by Vernon (1964) as the ‘halo and horns effects’. The halo effect became evident when students performed well during mentoring sessions with CEs, whereas the devil effect connotation had the opposite effect, which could be equally profound. In this study, the potential for bias seemed more of a halo effect, where CEs were inclined to consider how the student had performed during the clinical rotation, as opposed to only considering how the student performed during the assessment opportunity.

Our findings indicated that failing students during clinical performance assessments contributed to conflicting behaviours and emotions among participants in the relationship. Conflict between CEs and students resulted in friction and destabilisation of the T-L relationship and was not conducive to the promotion of learning. The findings confirmed that CEs were hesitant to fail students, as they tried to avoid traumatic experiences. The result was that they gave students the benefit of the doubt. Clinical educators’ indecisions, especially in borderline cases, could have far-reaching effects for the students involved, as well as for the profession. Unsafe practices could be extremely risky for students’ future practices and the profession, as confirmed in findings of previous studies by Duffy and Caledonian Nursing and Midwifery Research Centre (2003) and Hays (2008).

Some CEs acknowledged that students felt intimidated by their mere presence and perhaps their approach to facilitating learning, in which case they would be reluctant to ask questions, withdraw, distance themselves and thereby prevent self-disclosure. This could result in a lack of transparency and trust, which would have a negative effect on the relationship. During a study on nursing students’ perceptions, it was revealed by Lee, Cholowski and Williams (2002) that, although CEs stated that in practice they put students at the centre of learning, in actual fact they did not.

According to Parry (2004), power imbalances could exist in the T-L relationship, as was confirmed by this study. Feedback could result in a negative process if CEs were seen as ‘expert diagnosticians’ mentoring ‘attentive listeners’ (Parry 2004). These imbalances of power could lead to conflicting situations and could affect the learning process negatively (Ratner 2000), as some CEs found it challenging to provide feedback to students.

Similarly, dimensions of diversity present could affect the learning process and lead to conflict. Diverse cultural backgrounds, differences in language, personalities and learning preferences were present. Conflict could arise, which could affect the learning process negatively. Wood (2009) affirms that these could be impediments to learning, unless the ways in which education is presented to the group are appropriate for all.

There was a general consensus among CEs that the dual roles were challenging. Several CEs preferred the mentoring role to that of assessing. This is confirmed by Hays (2008), where some CEs also state their preference for not being involved in assessment practices, because of the fear of affecting the relationship with students negatively. Therefore, it is suggested that CEs should be allowed the choice of acting as mentors or assessors. Clinical educators highlighted the need for positive role models, support and an atmosphere free from tension as being important attributes in the clinical teaching context. Trust and respect for one another were essential. Only if such expectations were met would the T-L relationship be a positive one to ensure that learning took place.

Irrespective of some CEs preferring to act as both mentor and assessor to students, some acknowledged the presence of bias and inconsistencies among them.

It is, therefore, evident that for CEs to act as both mentor and assessor could affect the relationship with students in clinical education. Their interactions could, therefore, be challenging for both parties.

Furthermore, the findings revealed that solutions needed to be found for the specific challenges, expectations, preferences and limitations identified in this study in order to ensure a positive T-L relationship and the establishing of a learning paradigm, powered by communication between both parties as they work together as equal partners in communities of practice.

## Recommendations

It is essential to acknowledge the constraints of resources within the South African healthcare and educational systems when considering suggestions. There is a critical shortage of trained health personnel, overly full hospitals and more students being selected for training, thus requiring clinical experience (Kautzky & Tollman 2008; South Africa Council on Higher Education 2016). Within this context, the positioning theory (Davies & Harré 1990) is suggested to address the conflicts and challenges between CEs and students and to clarify the expectations of both CEs and students as described in this study. Using the positioning theory to reposition the students and CEs in the relationship towards a learning-centred paradigm, based on Vygotsky’s ZPD theory (Vygotsky & Cole 1978), could be a means to address the barriers mentioned in this study. This could be achieved through faculty development workshops with role play, demonstrating to CEs in practice how to reposition themselves in the T-L relationship where trust and clarity regarding expectations are fostered. If a community of practice could be established, a learning-centred paradigm would be created, which would be beneficial to students’ learning.

Tirado and Galvez (2007) believe that by the transformation of mentors’ and assessors’ actions, challenges and expectations can be resolved. Thus, this type of repositioning within the T-L relationship (Delany & Molloy 2009) between the participants should emphasise the process of learning.

Trust and respect for one another are essential in order for CEs to position themselves in relation to students towards a learning-orientated approach. Clinical educators would then be placed in relationship with students in the first place as a supportive mentor and secondly as a distanced assessor. ‘Both can mediate each other and be balanced’ (CE 3). It is a case of ‘putting on a different hat’ (CE 3). According to Higgs and McAllister (2006), high levels of efficiency occur when sufficient knowledge and experience have been gained. Clinical educators are then able to juggle the dual roles and even enjoy them. Fish (1998) describes a CE as a ‘professional artist’ who is an expert and can balance the demands, qualities and skills needed as a mentor and assessor like a tightrope walker (Higgs & McAllister 2006). Through this repositioning, a balance can, therefore, be found between the two roles and thereby re-establish the harmony in the T-L relationship.

## Conclusion

The study revealed that most CEs preferred to act as both mentors and assessors; however, the dual roles that CEs adopted could have a negative influence on the T-L relationship. These challenges were mainly the result of inconsistencies, bias and conflict arising when expectations of both CEs and students were unfulfilled.

However, challenges could be resolved by the formation of a ‘psychological symbiosis’ repositioning relationship in which the key components of mutual trust, respect and transparency are present. When the expectations of both parties are met, these anchors could lead to the transformation of the T-L relationship, and a learning-centred paradigm could be established, driven by open communication as both parties collaborate as equal partners in communities of practice. These communities of practice could be defined as ‘agents of change’ (Saint-Onge & Wallace 2003). Disparities within the relationship would be addressed and harmony would be re-established. Further studies should explore how workshops with role play, demonstrating to CEs in practice how to reposition themselves, impacts on the T-L relationship between CEs and students.

## References

[CIT0001] Borrell-Carrió F. & Epstein R.M., 2004, ‘Preventing errors in clinical practice: A call for self-awareness’, *Annals of Family Medicine* 2(4), 310–316. 10.1370/afm.80 15335129 PMC1466696

[CIT0002] Cruess S.R., Cruess R.L. & Steinert Y., 2008, ‘Role modelling – Making the most of a powerful teaching strategy’, *BMJ* 336(7646), 718–721. 10.1136/bmj.39503.757847.BE 18369229 PMC2276302

[CIT0003] Dalton M., Davidson M. & Keating J.L., 2012, ‘The assessment of physiotherapy practice (APP) is a reliable measure of professional competence of physiotherapy students: A reliability study’, *Journal of Physiotherapy* 58(1), 49–56. 10.1016/S1836-9553(12)70072-3 22341382

[CIT0004] Davies B. & Harré R., 1990, ‘Positioning: The discursive production of selves’, *Journal for the Theory of Social Behaviour* 20(1), 43–63. 10.1111/j.1468-5914.1990.tb00174.x

[CIT0005] Delany C. & Molloy E., 2009, *Clinical education in the health professions*, Elsevier Australia, Chatswood.

[CIT0006] Duffy K. & Caledonian Nursing & Midwifery Research Centre, 2003, *Failing students: A qualitative study of factors that influence the decisions regarding assessment of students’ competence in practice*, Caledonian Nursing and Midwifery Research Centre, Glasgow.

[CIT0007] Epstein R.M., Dannefer E.F., Nofziger A.C., Hansen J.T., Schultz S.H., Jospe N. et al., 2004, ‘Comprehensive assessment of professional competence: The Rochester experiment’, *Teaching and Learning in Medicine* 16(2), 186–196. 10.1207/s15328015tlm1602_12 15276897

[CIT0008] Ezzat A.M. & Maly M.R., 2012, ‘Building passion develops meaningful mentoring relationships among Canadian physiotherapists’, *Physiotherapy Canada* 64(1), 77–85. 10.3138/ptc.2011-07 23277688 PMC3280712

[CIT0009] Fish D., 1998, *Appreciating practice in the caring professions: Refocusing professional development and practitioner research*, Butterworth-Heinemann, Oxford.

[CIT0010] Gravett S. & Geyser H., 2004, *Teaching and learning in higher education*, Van Schaik, Pretoria.

[CIT0011] Harden R.M. & Crosby J., 2000, ‘The good teacher is more than a lecturer – The twelve roles of the teacher’, *AMEE Educational Guide* 20(4), 334–347.

[CIT0012] Hays R., 2008, ‘Assessment in medical education: Roles for clinical teachers’, *The Clinical Teacher* 5(1), 23–27. 10.1111/j.1743-498X.2007.00165.x

[CIT0013] Higgs J. & Mcallister L., 2006, ‘Being a clinical educator’, *Advances in Health Sciences Education* 12(2), 187–200. 10.1007/s10459-005-5491-2 17072770

[CIT0014] Kautzky K. & Tollman S.M., 2008, ‘A perspective on primary health care in South Africa’, *South African Health Review* 1, 17–30.

[CIT0015] Kelly M., 2009, ‘The role of theory in qualitative health research’, *Family Practice* 27(3), 285–290. 19875746 10.1093/fampra/cmp077

[CIT0016] Ker J., Cantillon P. & Ambrose L., 2008, ‘Teaching on a ward round’, *BMJ (British Medical Journal)* 337(12), 1930 10.1136/bmj.a1930 19050018

[CIT0017] Kilminster S., Cottrell D., Grant J. & Jolly B., 2007, ‘AMEE Guide No. 27: Effective educational and clinical supervision’, *Medical Teacher* 29(1), 2–19. 10.1080/01421590701210907 17538823

[CIT0018] Laitinen-Väänänen S., 2008, *The construction of supervision and physiotherapy expertise: A qualitative study of physiotherapy students’ learning sessions in clinical education*, University of Jyväskylä, Jyväskylä, Finland.

[CIT0019] Lave J. & Wenger E., 1991, *Situated learning: Legitimate peripheral participation*, Cambridge University Press, Cambridge.

[CIT0020] Lee W.-S.C., Cholowski K. & Williams A.K., 2002, ‘Nursing students’ and clinical educators’ perceptions of characteristics of effective clinical educators in an Australian university school of nursing’, *Journal of Advanced Nursing* 39(5), 412–420. 12175350 10.1046/j.1365-2648.2002.02306.x

[CIT0021] Lingham S. & Gupta R., 1998, ‘Mentoring for overseas doctors’, *British Medical Journal Classified* 317, 2–3.

[CIT0022] Maree K., 2007, *First steps in research,* Van Schaik, Pretoria.

[CIT0023] Meyer I.S., Louw A. & Erntzen D., 2017, ‘Physiotherapy students’ perceptions of the dual role of the clinical educator’, *SAJP*. 10.4102/sajp.v73i1.349 PMC6093108 30135902

[CIT0024] Miles M.B. & Huberman A.M., 1994, *Qualitative data analysis: An expanded sourcebook*, Sage, Thousand Oaks, CA.

[CIT0025] Neary M., 2000, ‘Supporting students’ learning and professional development through the process of continuous assessment and mentorship’, *Nurse Education Today* 20(6), 463–474. 10.1054/nedt.2000.0458 10959135

[CIT0026] Newton J.M., Billett S. & Ockerby C.M., 2009, ‘Journeying through clinical placements – An examination of six student cases’, *Nurse Education Today* 29(6), 630–634. 10.1016/j.nedt.2009.01.009 19231041

[CIT0027] Parry R., 2004, ‘Communication during goal setting in physiotherapy treatment sessions’, *Clinical Rehabilitation* 18, 668–682. 10.1191/0269215504cr745oa 15473119

[CIT0028] Power R. & Bogo M., 2003, ‘Educating field instructors and students to deal with challenges in their teaching relationships’, *The Clinical Supervisor* 21(1), 39–58. 10.1300/J001v21n01_04

[CIT0029] Ratner C., 2000, ‘Agency and Culture’, *Journal for the Theory of Social Behaviour* 30(4), 413–434. 10.1111/1468-5914.00138

[CIT0030] Saint-Onge H. & Wallace D., 2003, *Leveraging communities of practice for strategic advantage*, Butterworth-Heinemann, Burlington, MA.

[CIT0031] South Africa Council on Higher Education, 2016, ‘South African higher education reviewed; Two decades of Democracy’, *Che* 27(6), 795–808. 10.1080/02642060701453288

[CIT0032] Tilley D.S., Allen P., Collins C., Bridges A.N.N., Francis P. & Green A., 2007, ‘Using scaffolded instruction for practice-based learning’, *Journal of Professional Nursing* 23(5), 285–289. 10.1016/j.profnurs.2007.01.013 17903787

[CIT0033] Tirado F. & Galvez A., 2007, ‘Positioning theory and discourse analysis: Some tools for social interaction analysis’, *Forum: Qualitative Social Research* 8(2), 224–251.

[CIT0034] Van der Vleuten C.P.M. & Schuwirth L.W.T., 2005, ‘Assessing professional competence: From methods to programmes’, *Medical Education* 39(3), 309–317. 10.1111/j.1365-2929.2005.02094.x 15733167

[CIT0035] Vernon P.E., 1964, *Personality assessment: A critical survey*, Methuen, London.

[CIT0036] Vygotsky L.S. & Cole M., 1978, *Mind in society: The development of higher psychological processes*, Harvard University Press, Cambridge.

[CIT0037] Wass V., Van der Vleuten C., Shatzer J. & Jones R., 2001, ‘Assessment of clinical competence’, *Lancet* 357(9260), 945–949. 10.1016/S0140-6736(00)04221-5 11289364

[CIT0038] Wenger E., 1999, *Communities of practice*, Cambridge University Press, Cambridge.

[CIT0039] Wood W.B., 2009, ‘Innovations in teaching undergraduate biology and why we need them’, *Annual Review of Cell and Developmental Biology* 25(1), 93–112. 10.1146/annurev.cellbio.24.110707.175306 19575638

